# Gig work dependence and psychological distress in chronic illness

**DOI:** 10.1016/j.ssmph.2026.101927

**Published:** 2026-04-27

**Authors:** Zaiyue Wang, Lambert Zixin Li, Senhu Wang

**Affiliations:** a Renmin University of China, School of Population and Health, China; b National University of Singapore, Department of Management and Organisation, Singapore; c National University of Singapore, Department of Sociology and Anthropology, Singapore; d Tohoku University, Graduate School of Arts and Letters, Sendai, Japan

**Keywords:** Chronic illness, Gender, Gig work, Psychological distress, Unemployment

## Abstract

Chronic illness is associated with lower quality of life, yet it remains unclear whether this association differs by employment arrangements, particularly gig work. Using longitudinal data (2019–2024) from a nationally representative sample of 22,712 British workers, we examined how regular and gig employment moderate the association between chronic illness and psychological distress (GHQ-12; range: 0–12). Random-effects models indicate that individuals with chronic illness report 1.44 points higher psychological distress than those without such conditions (p < 0.001). This association is weaker among individuals in regular employment and low-dependence gig work, who report 0.64 and 0.56 points lower psychological distress, respectively, than those who are unemployed (p < 0.001). Although women with chronic illness experience greater psychological distress overall, among men with chronic illness, high-dependence gig work is associated with a 1.60-point increase in psychological distress relative to regular employment (p = 0.004), whereas no such pattern is observed among women. Taken together, the moderating role of gig work in health-related quality of life varies with workers’ dependence on gig work. Reducing the mental health burden of chronic illness may require access not only to employment, but also to high-quality jobs.

## Highlights

• Chronic illness relates to psychological distress, especially in women.
• Gig work relates to lower illness-related distress vs. unemployment.
• High gig dependence relates to higher distress vs. regular jobs.
• Adverse effects of high-dependence gig work are stronger in men.
• High-quality gig work may reduce mental health burden of illness.

## 1 Introduction

Chronic illness affects a growing share of the working-age population worldwide and is a major contributor to reduced health-related quality of life (Alonso et al., 2004). Beyond physical symptoms, individuals with chronic conditions face elevated risks of psychological distress, including anxiety and depression (Alonzo, 2000; Huang et al., 2023), which can compound disease burden and worsen long-term outcomes (Scott et al., 2016; Sporinova et al., 2019). Understanding the social conditions that mitigate or exacerbate the mental health consequences of chronic illness is therefore a central public health concern (Akif et al., 2024; Doherty & Gaughran, 2014). One such condition is employment (Facey & Eakin, 2010; Sorensen et al., 2021), yet whether and how employment shapes the mental health impact of chronic illness remains insufficiently understood.

A large body of research documents the mental health benefits of employment (Modini et al., 2016; Tausig, 2012). Employment provides not only income but also latent and manifest benefits such as time structure, social integration, identity, and a sense of purpose, all of which may buffer psychological distress (Jahoda, 1981). In contrast, unemployment is consistently associated with poorer mental health outcomes (Sterud et al., 2025), particularly among individuals with existing health vulnerabilities (Verhaak et al., 2005). However, this literature has largely treated employment as a binary condition (Paul et al., 2009; Wang, Ling, et al., 2022), overlooking substantial heterogeneity in employment arrangements and job quality (Cappelli & Keller, 2013; Cazes et al., 2015). As labor markets become increasingly fragmented (Benach et al., 2014; Kalleberg, 2009), this limitation is particularly salient.

Gig work and other forms of nonstandard employment have expanded rapidly over the past decade (Freni-Sterrantino & Salerno, 2021), offering flexibility and labor market access to individuals who may face barriers to traditional employment, including those with chronic illness who may be constrained into such work due to health-related limitations, discrimination, or reduced employer accommodation (Schur, 2002). For some, gig work may reduce the mental health burden of illness by enabling continued labor force participation (Lu et al., 2023). At the same time, gig work is often characterized by income volatility, job insecurity, weak social protections, and blurred work–life boundaries (Glavin & Schieman, 2022; Griesbach et al., 2019). These features may undermine mental health, especially when individuals depend heavily on gig work as their primary source of income (Ravenelle, 2019; Wang, Li, & Coutts, 2022). Whether gig work functions as a protective or harmful context for the mental health of people with chronic illness therefore remains an open empirical question.

In addition, the mental health consequences of employment arrangements may differ systematically by gender. Prior research suggests that women with chronic illness experience greater psychological distress overall (Laurin et al., 2007; Tovt-Korshynska et al., 2001), reflecting gendered patterns of caregiving, labor market inequality, and health-related stigma (Bird & Rieker, 1999; Bracke et al., 2020; Thakral et al., 2019). Conversely, men may be particularly vulnerable to the psychological costs of precarious employment when gig work threatens norms of economic stability and breadwinning (Kan & Laurie, 2018; Warren, 2022). Yet few studies have examined how gender intersects with employment type to shape the mental health burden of chronic illness.

This study addresses these gaps by examining how different forms of employment moderate the association between chronic illness and psychological distress, and whether these patterns vary by gender. Using longitudinal data from 2019 to 2024 drawn from a nationally representative sample of British workers, we compare distinct employment arrangements, including regular employment, gig work, and unemployment, and further distinguish between low- and high-dependence gig work (Guo et al., 2025). Leveraging a nationally representative sample enhances the external validity of the analyses, while the longitudinal design allows us to more rigorously assess within-individual changes over time, strengthening internal validity.

Our analyses show that chronic illness is associated with higher psychological distress, especially among women, but that this association is weaker among individuals engaged in regular employment or gig work relative to unemployment. However, among men with chronic illness, psychological distress is higher among those with high dependence on gig work than among those in regular employment, underscoring the importance of job quality and stability (De Moortel et al., 2025).

This study contributes to research on social determinants of health by systematically examining employment as a critical but underexplored determinant of health-related quality of life among people with chronic illness (Aronsson & Huijts, 2025). Importantly, we move beyond binary distinctions between employment and unemployment by differentiating among forms of employment, recognizing that individuals with chronic illness may have limited access to standard jobs and may be disproportionately represented in nonstandard works (Cropanzano et al., 2023). By focusing on employment quality rather than employment status alone (Cazes et al., 2015), this study advances understanding of how labor market arrangements function as social determinants of mental health among people with chronic illness. In addition, our findings highlight the role of gender norms surrounding illness, work, and economic responsibility in shaping mental health outcomes (Bird & Rieker, 1999; Lennon, 1987). By showing that gender moderates the relationship between chronic illness and mental health, and that employment type matters differently within gender groups, this study suggests that research on social determinants of health should more explicitly consider previously neglected subgroup heterogeneity.

From a policy perspective, our findings suggest that employment has the potential to reduce the mental health burden of chronic illness, indicating that labor market interventions may be meaningfully integrated into health policy (Drake & Wallach, 2020). At the same time, the results underscore that the benefits of employment depend on job quality, suggesting that policy efforts should extend beyond reemployment per se to improving access to stable, high-quality jobs (Baekgaard et al., 2024; De Moortel et al., 2025). Finally, the observed gender differences point to the need for targeted policy responses: while female patients with chronic illness may require enhanced mental health support, male patients in precarious or high-dependence gig work conditions may also represent a vulnerable group warranting focused intervention.

## 2 Theory and hypotheses

### 2.1 Chronic illness and psychological distress

Chronic illness or disability refers to long-standing physical or mental health conditions that limit daily functioning or require ongoing management (Charmaz, 2000). As disease profiles shift from acute to chronic conditions, chronic illness has become increasingly prevalent among working-age adults (Hajat & Stein, 2018). Psychological distress, capturing symptoms such as anxiety, depression, and emotional strain, is a core dimension of health-related quality of life and a central concern in public health (Ridner, 2004), given its effects on physical disease progression, healthcare utilization, labor market outcomes, and societal burden (Müller et al., 2021; Scott et al., 2016; Sporinova et al., 2019). Consistent with stress theory, chronic illness represents a persistent stressor that is expected to elevate psychological distress through multiple pathways, including ongoing physical symptoms, functional limitations, uncertainty about future health, increased financial strain, and experiences of stigma or social withdrawal (Akif et al., 2024; Doherty & Gaughran, 2014; Heyworth et al., 2009).

Hypothesis 1 (H1). Chronic illness is associated with higher psychological distress.

### 2.2 Employment as a buffer: latent deprivation theory

Employment is a fundamental social determinant of mental health. Beyond providing income, employment supplies latent benefits that are essential for psychological well-being, including time structure, social integration, collective purpose, identity, and regular activity (Paul et al., 2009). According to Jahoda's latent deprivation theory (Jahoda, 1981), unemployment undermines mental health by depriving individuals of these psychosocial resources, whereas employment restores them. For individuals with chronic illness, employment may be especially protective because illness often disrupts daily routines, social participation, and role identity (Harpur & Blanck, 2020). In this context, employment can counteract illness-related isolation and loss of control, while unemployment may compound distress by reinforcing uncertainty, dependency, and social exclusion (Jahoda, 1982). Importantly, this framework speaks most directly to regular employment and does not imply that all employment arrangements provide equivalent mental health benefits.

Hypothesis 2 (H2). Unemployment positively moderates the association between chronic illness and psychological distress, such that the association is stronger among unemployed individuals than among those in regular employment.

### 2.3 Gig work as a distinct and heterogeneous employment arrangement

Employment, however, is not a homogeneous condition. While latent deprivation theory posits that employment is generally beneficial to mental health by restoring income and psychosocial resources, the Job Demands–Resources model and broader job quality frameworks emphasize that different forms of employment may entail uneven demands and resources (Demerouti et al., 2001; Cazes et al., 2015), with divergent implications for psychological distress. One increasingly salient source of such heterogeneity in contemporary labor markets is gig work.

Gig work refers to platform-mediated, task-based work typically performed outside standard employment relationships (Cropanzano et al., 2023). It has expanded rapidly and may be particularly relevant for people with chronic illness, who often face barriers to regular employment due to health constraints, limited workplace accommodation, or discrimination (Penner et al., 2025; Harpur & Blanck, 2020). Its flexibility in scheduling and workload may facilitate continued labor force participation despite fluctuating health (Donovan et al., 2016). At the same time, gig work varies substantially in stability, security, and income dependence, making its mental health implications theoretically ambiguous rather than uniformly beneficial or harmful (Berger et al., 2019; Lu et al., 2023).

To capture this heterogeneity, we distinguish between low-dependence gig work, in which individuals combine gig work with other non-platform employment, and high-dependence gig work, in which individuals rely exclusively on gig work as their primary source of income (Schor et al., 2020; Guo et al., 2025). This distinction reflects meaningful differences in exposure to job insecurity, income volatility, and access to non-platform resources (Glavin & Schieman, 2022), which are central to both JD–R and job quality perspectives.

#### 2.3.1 Low-dependence gig work: job resources, flexibility, and stress buffering

The Job Demands–Resources (JD–R) model posits that mental health depends on the balance between job demands and available resources (Demerouti et al., 2001). Low-dependence gig work may attenuate the mental health burden of chronic illness because it combines flexibility with resource diversification. Individuals in low-dependence gig work typically retain income stability, benefits, and organizational support from non-platform jobs, while using gig work to supplement earnings or adjust work intensity around health needs (Inanc, 2018; Milkman et al., 2021). For people with chronic illness, this configuration may increase perceived control over work pace, reduce financial strain, and allow continued role engagement without full exposure to platform precarity (Lu et al., 2023).

Hypothesis 3 (H3). Low-dependence gig work negatively moderates the association between chronic illness and psychological distress, such that the association is weaker among low-dependence gig workers than among those in regular employment.

#### 2.3.2 High-dependence gig work: job demands, precarity, and resource depletion

In contrast, high-dependence gig work may exacerbate psychological distress among individuals with chronic illness. High-dependence gig workers face substantial job demands, including income instability, algorithmic control, weak social protection, and constant pressure to secure tasks (Benach et al., 2014; Wang, Ling, et al., 2022; Wood et al., 2019). From both the JD–R and Conservation of Resources perspectives, reliance on gig work as a sole income source exposes workers to chronic resource threat and loss (Halbesleben et al., 2014). For individuals with chronic illness, who already experience depleted physical and psychological resources, these demands may intensify stress, anxiety, and emotional exhaustion rather than buffer them.

Hypothesis 4 (H4). High-dependence gig work positively moderates the association between chronic illness and psychological distress, such that the association is stronger among high-dependence gig workers than among those in regular employment.

### 2.4 Gender differences in psychological distress by chronic illness and high-dependence gig work

The mental health implications of chronic illness vary by gender. A large literature shows that women report higher psychological distress in response to health-related stressors, reflecting gendered patterns of caregiving responsibility, labor market inequality, and social expectations around emotional labor and health (Thakral et al., 2019; Gove, 1984; Caputo & Simon, 2013). Women with chronic illness may therefore experience greater cumulative strain as illness interacts with work and family roles (Bird & Rieker, 1999).

Hypothesis 5 (H5). The association between chronic illness and psychological distress is stronger among women than among men.

Moreover, there may be gender differences in the moderating role of high-dependence gig work in psychological distress from chronic illness. In this study, we focus on how this moderation manifests within each gender group. Social norms emphasizing men's role as primary economic providers suggest that income instability and employment precarity may be particularly salient sources of psychological strain for men (Kan & Laurie, 2018; Warren, 2022). When chronic illness constrains work capacity and individuals rely heavily on gig work that does not provide stable earnings or security, psychological distress may be more strongly evident among men (Kim et al., 2008; Marler & Moen, 2005). By contrast, the same pattern may be less pronounced or absent among women.

Hypothesis 6 (H6). The positive moderation of the association between chronic illness and psychological distress by high-dependence gig work (H4) is expected to be present among men but not among women.

## 3 Methods

### 3.1 Data and sample

This study draws on data from Wave 10 (2018-2020), Waves 11 (2019–2021), 12 (2020–2022), 13 (2021–2023), and 14 (2022–2024) of the UK Household Longitudinal Study (UKHLS). Wave 11 is the first wave to include detailed questions identifying gig work participation, enabling the analysis of how different employment arrangements moderate the association between chronic illness and psychological distress. Information of chronic illness is measured in the Wave 10, while employment arrangements and psychological distress are measured in subsequent waves. The UKHLS employs a multistage stratified and clustered sampling design to obtain a nationally representative sample of more than 40,000 UK households. Response rates range from 45.2% to 79.4% across waves, and attrition rates range from 4.13% to 9.78% (see Table A1 and A2 in Appendix for details).

We restrict the analytic sample to respondents who participated in at least two of the four selected waves. We exclude individuals for whom chronic illness information is missing and those for whom employment arrangement classification is not applicable, including full-time students and individuals on maternity leave, as well as respondents with missing values on key variables. After applying these criteria, the final analytic sample consists of 22,712 workers and 75,428 person–wave observations. Details of sample construction are provided in Table A3.

### 3.2 Variables and measures

The dependent variable is psychological distress, measured using the 12-item General Health Questionnaire (GHQ-12), a widely validated instrument assessing symptoms such as anxiety, depression, reduced happiness, and sleep disturbance (Goldberg & Hillier, 1979). Each item is rated on a four-point scale ranging from 1 (“better than usual”) to 4 (“much worse than usual”). Following standard practice, responses indicating worse-than-usual mental health are coded as 1 and those indicating usual or better-than-usual mental health are coded as 0 (Li & Wang, 2022). Summing the 12 items yields a score ranging from 0 to 12, with higher values indicating greater psychological distress (Cronbach's α = 0.90).

The key independent variable is chronic illness, operationalized using the UKHLS measure of long-standing illness or disability. This binary indicator equals 1 if respondents report a health condition or disability that causes substantial difficulty in one or more domains of daily functioning, including mobility, sensory functioning, cognition, communication, or self-care, and 0 otherwise.

The moderator is employment arrangement, classified into four categories: regular employment, low-dependence gig work, high-dependence gig work, and unemployment. High-dependence gig work refers to individuals engaged exclusively in platform-based work, whereas low-dependence gig work includes individuals who combine gig work with other non-platform employment.

Control variables include gender, age, race partnership status, presence of dependent children in the household, and educational attainment. Wave fixed effects are included to account for time-specific influences. Variable definitions and coding are detailed in Table A4. Detailed information about how this study measures two types of gig work and the correspondence to other literature are reported in Table A5.

### 3.3 Analytic strategy

First, we conduct descriptive analyses, including ANOVA and chi-square tests, to examine differences in psychological distress and sociodemographic characteristics across health status, employment arrangements, and gender.

Second, we estimate random-effects regression models to examine the association between chronic illness or disability and psychological distress. Chronic illness is measured in the preceding survey wave (Wave 10) and included as a lagged independent variable to predict psychological distress in subsequent waves, which helps establish temporal ordering and reduces concerns about reverse association. Random-effects models are appropriate because the primary independent variable is lagged and largely time-invariant and because the analysis focuses on both within- and between-individual variation (Clark & Linzer, 2015). Analyses are also conducted separately for men and women to examine gender differences.

Third, we include interaction terms between employment arrangement and chronic illness or disability, using regular employment as the reference category. These models assess whether the association between chronic illness and psychological distress differs across employment arrangements relative to regular employment. To examine gender-specific patterns, these interaction models are further estimated separately for men and women.

## 4 Results

Table 1 presents descriptive statistics for the key variables. Individuals with chronic illness exhibit substantially higher levels of psychological distress than those without chronic illness. Among men with chronic illness, the mean psychological distress score is 2.38, compared with 1.21 among men without chronic illness. A similar pattern holds for women, with an average score of 3.24 for those with chronic illness versus 1.85 for those without. These descriptive patterns are consistent with Hypothesis 1 (H1). Employment arrangements also differ markedly by health condition: individuals with chronic illness are far more likely to be out of paid employment, whereas regular employment is much more common among those without chronic illness (approximately 62–64%) than among those with chronic illness (about 36%). Across health conditions, women report higher psychological distress than men, with women with chronic illness exhibiting the highest distress levels. In terms of employment patterns, women are slightly more represented in low-dependence gig work, whereas men are modestly more represented in high-dependence gig work.

**Table 1:** Descriptive statistics of key variables.

	With chronic illness		Without chronic illness		F/χ2
	Men	Women	Men	Women	
Psychological distress, M (SD)	2.38 (3.52)	3.24 (3.92)	1.21 (2.51)	1.85 (3.09)	P < 0.001
Age, M (SD)	60.58 (16.13)	57.17 (15.73)	51.56 (16.31)	49.37 (15.62)	P < 0.001
Educational attainment, %					P < 0.001
Below tertiary	65.01	62.26	50.38	48.78	
Tertiary	34.99	37.74	49.62	51.22	
Race, %					P < 0.001
White	85.59	82.33	82.62	80.20	
Non-white	14.41	17.67	17.38	19.80	
Presence of dependent children, %					P < 0.001
Yes	17.41	22.93	34.01	36.85	
No	82.59	77.07	65.99	63.15	
Partnership status, %					P < 0.001
Yes	85.59	75.42	85.82	78.86	
No	14.41	24.58	14.18	21.14	
Employment arrangement, %					P < 0.001
Unemployment	60.70	60.14	29.97	33.01	
Regular employment	36.26	36.56	64.01	62.16	
High-dependence gig work	0.30	0.20	0.27	0.25	
Low-dependence gig work	2.74	3.10	5.75	4.58	
N	8094	10521	25860	30953	75428
Row %	10.73	13.95	34.28	41.04	100

Note: % = Proportion, M = Mean, SD = Standard deviation; N = Number of respondents.

Table 2 reports random-effects regression results examining the association between chronic illness and psychological distress. Across all models, chronic illness is associated with higher psychological distress. In the full-sample model (Model 1), individuals with chronic illness score on average 1.439 points higher on psychological distress than those without such conditions (p < 0.001), providing support for Hypothesis 1 (H1).

**Table 2:** Random-effects regression on chronic illness and psychological distress.

	Model 1	Model 2	Model 3	Model 4
	Total	Total	Men	Women
Chronic illness (ref. = Without chronic illness)				
With chronic illness	1.439∗∗∗	1.511∗∗∗	1.348∗∗∗	1.508∗∗∗
	(0.039)	(0.051)	(0.053)	(0.056)
Gender (ref. = Female)				
Male	−0.580∗∗∗	−0.539∗∗∗		
	(0.033)	(0.038)		
Chronic illness ∗ Gender (ref. = Female)		−0.166∗		
		(0.076)		
Partnership status (ref. = No)				
Yes	−0.380∗∗∗	−0.378∗∗∗	−0.072	−0.535∗∗∗
	(0.041)	(0.041)	(0.066)	(0.054)
Race (ref. = Non-white)				
White	0.160∗∗∗	0.161∗∗∗	0.087	0.218∗∗∗
	(0.042)	(0.042)	(0.058)	(0.060)
Age	−0.030∗∗∗	−0.030∗∗∗	−0.030∗∗∗	−0.031∗∗∗
	(0.001)	(0.001)	(0.002)	(0.002)
Presence of dependent children (ref. = No)				
Yes	0.259∗∗∗	0.258∗∗∗	0.292∗∗∗	0.195∗∗∗
	(0.035)	(0.035)	(0.049)	(0.049)
Educational attainment (ref. = Below tertiary)				
Tertiary	0.188∗∗∗	0.188∗∗∗	0.271∗∗∗	0.110∗
	(0.033)	(0.033)	(0.044)	(0.048)
Employment arrangement (ref. = Unemployment)				
Regular employment	−0.635∗∗∗	−0.635∗∗∗	−0.706∗∗∗	−0.607∗∗∗
	(0.033)	(0.033)	(0.047)	(0.046)
High-dependence gig work	0.243	0.243	0.373	0.119
	(0.188)	(0.188)	(0.242)	(0.282)
Low-dependence gig work	−0.560∗∗∗	−0.561∗∗∗	−0.689∗∗∗	−0.476∗∗∗
	(0.055)	(0.055)	(0.073)	(0.080)
Within R-squared	0.005	0.005	0.005	0.006
Observations	75428	75428	33954	41474
Number of respondents	22712	22712	10121	12591

Note: Standard errors are in parentheses. ∗∗∗p < 0.001, ∗∗p < 0.01, ∗p < 0.05; ref. = reference category.

Employment arrangement is also associated with psychological distress. Compared with individuals not engaged in paid work, those in regular employment report significantly lower psychological distress (β = −0.635, p < 0.001). Low-dependence gig work is likewise associated with lower psychological distress (β = −0.560, p < 0.001), a pattern that holds for both men and women. In contrast, high-dependence gig work does not exhibit a statistically significant main association with psychological distress, both for total sample (β = 0.243, p = 0.195) and samples by gender (β = 0.373, p = 0.123; β = 0.119, p = 0.673).

Table 3 presents the differences by employment arrangements in the association between chronic illness and psychological distress. In the full-sample model (Model 1), the association between chronic illness and psychological distress is stronger among unemployed individuals than among those in regular employment, and this difference is statistically significant (interaction coefficient = 0.398, p < 0.001), thereby supporting Hypothesis 2 (H2). Meanwhile, the association between chronic illness and psychological distress is weaker among individuals in low-dependence gig work than among those in regular employment (interaction coefficient = −0.258, p = 0.039), providing support for Hypothesis 3 (H3). High-dependence gig work shows a positive interaction coefficient (interaction coefficient = 0.811), indicating a stronger association between chronic illness and psychological distress, although this estimate does not reach statistical significance in the full sample (p = 0.064), providing partial support for Hypothesis 4 (H4).

**Table 3:** Moderating effect of employment arrangement.

Chronic illness ∗ Employment arrangement (ref. = Regular employment)	Model 1	Model 2	Model 3
	Total	Men	Women
With chronic illness ∗ Unemployment	0.398∗∗∗	0.310∗∗∗	0.457∗∗∗
	(0.068)	(0.093)	(0.097)
With chronic illness ∗ High-dependent gig work	0.811	1.599∗∗	−0.025
	(0.438)	(0.551)	(0.676)
With chronic illness ∗ Low-dependent gig work	−0.258∗	−0.168	−0.339
	(0.125)	(0.174)	(0.176)
Within R-squared	0.005	0.006	0.006
Observations	75428	33954	41474
Number of respondents	22712	10121	12591

Note: Standard errors are in parentheses. ∗∗∗p < 0.001, ∗∗p < 0.01, ∗p < 0.05; ref. = reference category. Controlled for all covariates mentioned in Table 2.

Gender-stratified models in Table 2 show that the association of chronic illness and psychological distress is larger among women (β = 1.508) than among men (β = 1.348), and the interaction between chronic illness and gender is statistically significant (p = 0.028), supporting Hypothesis 5 (H5). Further gender-stratified models in Table 3 indicate that the moderating role of employment arrangement differs across subgroups. Among men (Model 3), the association between chronic illness and psychological distress is stronger for those dependent on gig work (interaction coefficient = 1.599, p = 0.004), whereas among women (Model 4), the corresponding interaction is small and not statistically significant (β = −0.025, p = 0.971). These results suggest that the moderating role of high-dependence gig work is present among men but not observed among women, consistent with Hypothesis 6 (H6).

**Fig. 1:**
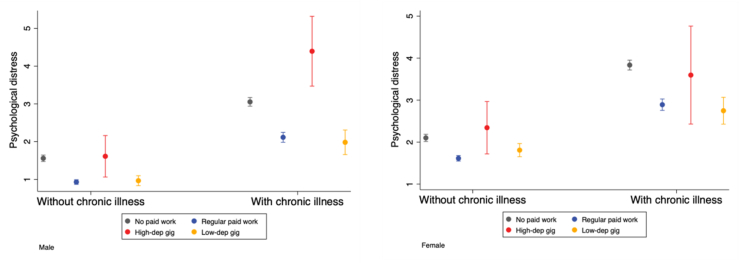
Moderating effect of employment arrangement (by gender).

Fig. 1 illustrates these patterns. While individuals with chronic illness generally report higher psychological distress across all employment arrangements, the gap is most pronounced among men engaged in high-dependence gig work, whereas no comparable pattern is observed among women.

To ensure the validity of the results, we further conduct two robustness checks. First, we change an alternative definition for gig works. We redefine high-dependence gig work as the proportion of gig work hours surpass 50% in total work hours. Table A6 (appendix) shows the baseline results using this alternative classification of employment arrangements. Chronic illness is positively associated with psychological distress in whole sample as well as in gender-stratified models, which is consistent with the main analysis. Moreover, the gender difference also remains unchanged, with women exhibiting a stronger association between chronic illness and psychological distress than men. Table A7 (appendix) shows the differences by employment arrangements under the alternative classification of gig works. Results show that, relative to regular employment, the association between chronic illness and psychological distress is stronger among unemployed individuals. By contrast, this association is weaker among those in low-dependence gig work, whereas it is stronger among those in high-dependence gig work. Notably, this pattern remains statistically significant among men, further supporting the gender differences observed in the main analysis.

Second, we use individuals with health problems lasting at least 12 months as an alternative independent variable. Table A8 (appendix) shows the baseline association between health problems and psychological distress and the gender difference are consistent using this measure. Table A9 (appendix) shows the moderation analyses. Although it cannot indicate a moderating role of low-dependence gig work, as the interaction coefficient between low-dependence gig work and health problems does not reach statistical significance (β = 0.007, p = 0.945), the subgroup differences by other two employment arrangements remain unchanged and the results from gender-stratified models also persist.

## 5 Discussion

### 5.1 Summary of key findings

This study examined how employment arrangements moderate the association between chronic illness and psychological distress, using longitudinal data from a large, nationally representative sample of British workers. Moving beyond binary distinctions between employment and unemployment, we differentiated among regular employment, low-dependence gig work, and high-dependence gig work, and further assessed whether these patterns vary by gender. By integrating perspectives from stress theory, latent deprivation theory, and job demands–resources frameworks, the study provides a more nuanced understanding of how health-related quality of life differs by labor market arrangements.

Consistent with a large body of prior research (Akif et al., 2024; Doherty & Gaughran, 2014), we find that chronic illness or disability is associated with higher psychological distress. This pattern holds for both men and women and is consistent with stress-based accounts emphasizing the psychological burden of persistent symptoms, functional limitations, uncertainty, and stigma (Alonzo, 2000). We also find that the association between chronic illness and psychological distress is stronger among women than among men, echoing previous evidence that women experience greater psychological distress in response to health-related stressors (Tovt-Korshynska et al., 2001; Laurin et al., 2007). These findings reaffirm that chronic illness remains a major public health challenge not only because of its physical consequences but also because of its substantial mental health burden (Alonso et al., 2004).

Beyond these established patterns, the study yields several novel findings. First, we show that the association between chronic illness and psychological distress depends critically on employment arrangements. Individuals with chronic illness who are engaged in regular employment experience substantially lower psychological distress than those who are unemployed, consistent with latent deprivation theory (Jahoda, 1981). Employment appears to buffer the mental health burden of chronic illness by restoring income, social integration, daily structure, and role identity, resources that are often disrupted by illness (Baekgaard et al., 2024). This finding underscores employment as an important social determinant of health-related quality of life among people with chronic conditions (Drake & Wallach, 2020).

Second, and more importantly, we demonstrate that not all forms of employment provide equivalent mental health benefits. By distinguishing between low- and high-dependence gig work, we show that gig employment is internally heterogeneous in its association with psychological distress (Guo et al., 2025). Low-dependence gig work, when combined with other non-platform employment, is associated with lower psychological distress relative to unemployment and may attenuate the association between chronic illness and distress. This pattern suggests that flexible, supplementary gig work may function as a coping resource for people with chronic illness, allowing them to remain economically and socially engaged while retaining access to income stability and protections from non-platform jobs (Inanc, 2018; Milkman et al., 2021). In contrast, high-dependence gig work is associated with greater psychological distress relative to regular employment and may amplify the mental health burden of chronic illness. This finding aligns with job demands–resources and job quality perspectives, which emphasize the psychological costs of income volatility, job insecurity, and weak social protections when gig work becomes a primary livelihood (Benach et al., 2014; Wood et al., 2019).

Third, we identify an important and previously underexplored gender heterogeneity. While the chronic illness–distress association is not stronger among women who depend on gig work, the association is stronger among men. This pattern suggests that employment arrangements are embedded within gendered social contexts (Matud, 2004). High-dependence gig work may be particularly psychologically taxing for men with chronic illness when precarious employment threatens economic stability and paid-work identities that are socially salient for men (Kim et al., 2008). This finding highlights that employment is not only a socioeconomic institution but also a gendered one, with differential mental health implications across gender groups (Ensminger & Celentano, 1990).

### 5.2 Theoretical and policy implications

Together, these findings make several theoretical contributions. First, the study advances research on social determinants of health by demonstrating that employment status moderates health-related quality of life among people with chronic illness. Second, and most centrally, it shows that employment arrangements, and not employment per se, are critical for understanding mental health inequalities in contemporary labor markets. By differentiating between low- and high-dependence gig work, the study clarifies why prior research on gig work and mental health has yielded mixed results (Berger et al., 2019; Freni-Sterrantino & Salerno, 2021; Lu et al., 2023; Wang, Li, & Coutts, 2022). Third, the findings contribute to the literature on gender and health by showing that the mental health implications of precarious employment are gender-specific, reinforcing the need to treat employment as a gendered social institution in public health research (Hammarström, 2021).

The findings also have clear policy implications. Policies aimed at improving the well-being of people with chronic illness should not focus solely on labor force participation (Baekgaard et al., 2024), but also on the quality and stability of available employment arrangements (De Moortel et al., 2025). While facilitating access to flexible work opportunities may benefit some individuals with chronic illness, reliance on precarious, high-dependence gig work may exacerbate psychological distress, particularly among men. Public health and labor market policies should therefore promote access to stable, high-quality jobs and extend social protections, such as income security, health benefits, and worker protections, to those engaged in nonstandard employment (Singh et al., 2025; Sorensen et al., 2021). Gender-sensitive approaches may be especially important, given the differential mental health consequences observed across men and women.

### 5.3 Limitations and strengths

Several limitations should be acknowledged. First, chronic illness or disability is measured as a binary indicator and does not capture specific diagnoses, severity, or duration of conditions, which may mask important heterogeneity. In addition, while our classification of gig work into low- and high-dependence categories follows established practice, it cannot fully capture variation in job quality across platforms or tasks (Guo et al., 2025). Second, psychological distress is self-reported and may be subject to reporting biases, including social desirability and recall error, highlighting the need for more objective measures of health (Chandola et al., 2019). Third, since random-effects estimates may be biased if unobserved individual characteristics are correlated with the explanatory variables, the results in our study cannot be interpreted as strict causality. Finally, although differential attrition may occur if respondents with chronic illness are more likely to drop out of the panel, attrition rates are relatively low across waves and are unlikely to materially affect the study's main conclusions.

Despite these limitations, the study has several notable strengths. It is among the few studies in this area to use longitudinal data, which helps reduce bias from time-invariant unobserved factors such as personality or baseline mental health (Scott et al., 2016). In addition, the use of a large, nationally representative probability sample enhances the generalizability of the findings, addressing a key limitation of much prior research on gig work and health (Glavin & Schieman, 2022).

### 5.4 Directions for future research

Future research could build on these findings by examining specific chronic conditions, exploring platform-level differences in gig work quality (Wood et al., 2025), and assessing longer-term mental health trajectories. Further attention to intersectional heterogeneity, such as education, race, or caregiving responsibilities (Sullivan, 2019), may also deepen understanding of how employment arrangements shape health inequalities.

## 6 Conclusion

In conclusion, this study shows that the mental health burden of chronic illness varies not only by whether individuals are employed, but by how they are employed. The association between chronic illness and psychological distress differs by employment arrangements and gender, and the moderating role of gig work varies by whether workers depend on it. Therefore, public health and labor policies may address social inequality through improvements in job quality in an increasingly fragmented world of work.

## CRediT authorship contribution statement

**Zaiyue Wang:** Writing – original draft, Methodology, Formal analysis. **Lambert Zixin Li:** Writing – original draft, Conceptualization. **Senhu Wang:** Writing – review & editing, Methodology, Conceptualization.

## Declaration of generative AI use

None.

## Ethical statement

The manuscript contains secondary analysis of anonymized data only and does not require an ethical approval.

## Financial disclosure statement

Dr. Wang is funded by Ministry of Education, Singapore, Tier 1 Grant (A-8002445-00-00). Dr. Li is funded by National University of Singapore and National Natural Science Foundation of China (72574210). The funders had no role in the design, conduct, or interpretation of the study.

## Declaration of interest statement

All authors declare no competing interests.

## Data Availability

Data will be made available on request.
