# Supplementary material for: Gig work dependence and psychological distress in chronic illness
Source: SSM Popul Health. 2026 Apr 27;34:101927. doi: 10.1016/j.ssmph.2026.101927 (PMC13147377; doi:10.1016/j.ssmph.2026.101927)
Supplement: Multimedia component 1 [file mmc1.docx]

**Online Appendix**

| **Table A1**  **Response Rates Across Waves in the UKHLS (%)** | |
| --- | --- |
| **Wave** | **Response rate (%)** |
| Wave 10 | 52.2 - 79.4 |
| Wave 11 | 53.0 - 78.9 |
| Wave 12 | 45.2 - 78.3 |
| Wave 13 | 46.6 - 76.4 |
| Wave 14 | 49.3 - 76.6 |
| Note: Response rates are calculated as the proportion of eligible sample members who completed an interview among those issued to fieldwork in each wave. The reported ranges reflect variation across multiple samples (e.g., The General Population Sample, The Ethnic Minority Boost Sample, etc.), as documented in the UKHLS Main Survey User Guide. | |

| **Table A2**  **Attrition and Retention Rates Across Waves, Overall and by Chronic Illness Status** | | | |
| --- | --- | --- | --- |
| **Wave** | **Overall** | **With Chronic Illness** | **Without Chronic Illness** |
|  | Retention (Attrition), % | Retention (Attrition), % | Retention (Attrition), % |
| Wave 11 | 100.00 (0.00) | 100.00 (0.00) | 100.00 (0.00) |
| Wave 12 | 95.86 (4.14) | 95.82 (4.18) | 95.88 (4.12) |
| Wave 13 | 92.84 (7.16) | 91.53 (8.47) | 93.27 (6.73) |
| Wave 14 | 90.22 (9.78) | 88.28 (11.72) | 90.85 (9.15) |

| **Table A3**  **Steps of analytic sample construction** | |
| --- | --- |
| **Sample description** | **Sample** |
| Original sample of United Kingdom Household Longitudinal Study (waves 11, 12, 13, and 14) | N = 124,748 |
| Restrict the sample to individuals who have participated at least two waves | N = 110,945 |
| Restrict the sample to individuals who are full-time students or on maternity leave | N = 105,177 |
| Restrict the sample to respondents who completed all key questions (excluding those who are not asked or not applicable) and delete individuals with missing values for the variables used | N = 75,428 |

| **Table A4**  **Variable, definition, and coding** | | |
| --- | --- | --- |
| **Variable** | **Definition** | **Coding** |
| Dependent variable |  |  |
| Psychological distress | Measured by the 12-item General Health Questionnaire (GHQ-12), a validated scale capturing depression, anxiety, happiness, and sleeping problems. Responses are coded on a four-point scale (1 = better than usual; 4 = much less than usual), with higher values indicating poorer psychological health. | Continuous |
| Independent variable |  |  |
| Chronic illness | Whether the respondent reports having a chronic illness. | 1 = Yes (with chronic illness), 0 = No |
| Moderator variable |  |  |
| Employment arrangement |  |  |
| Unemployment | Respondents who reported not engaging in any form of paid employment, including both conventional and gig economy work, during the reference period. | 0 = Unemployment |
| Regular employment | Respondents who engaged in paid employment or self-employment not involving any platform-based (gig) activities. | 1 = Regular employment |
| High-dependence gig work | Gig work is identified based on UKHLS survey questions asking whether respondents have undertaken any platform-mediated work via websites, apps, or online platforms. Specifically, respondents are asked whether they have engaged in the following types of work, including carrying passengers (e.g., taxi or ride-hailing services), delivering food and drink, providing courier services and other manual (e.g., cleaning, plumbing, building) or non-manual (e.g., software development, translation, legal advice, accounting services) tasks.  Respondents who reported engaging in at least one type of gig work and did not report holding any other job are classfied as high-dependence gig work | 2 = High-dependence gig work |
| Low-dependence gig work | Respondents who engaged in gig economy activities, as defined above, in combination with other forms of paid employment. | 3 = Low-dependence gig work (gig jobs + other paid work) |
| Control variable |  |  |
| Age | Respondent’s age in years at the time of survey interview. Treated as a continuous, time-varying measure. | Continuous |
| Gender | Respondent’s self-reported gender. | 0 = Female; 1 = Male |
| Partnership status | Respondent’s current legal or cohabitation status. | 0 = No; 1 = Yes |
| Presence of dependent children | Whether there are any dependent children living in the respondent’s household. | 0 = No; 1 = Yes |
| Race | Respondents’ self-identified racial or ethnic background. | 0 = Non-White; 1 = White |
| Educational attainment | Respondents’ highest educational attainment | 0 = Below tertiary; 1 = Tertiary |

| **Table A5**  **Detailed information about measures of gig work** | | | | |
| --- | --- | --- | --- | --- |
| **Survey wave** | **Original survey item (UKHLS)** | **Response options (original coding)** | **Recoding in this study** | **Correspondence to literature** |
| Waves 11–12 | Thinking about the past month, which, if any, of the following have you done in order to make money using a website, platform or app? (CODE ALL THAT APPLY) | 1. Carried passengers in your vehicle (e.g. taxi rides)  2. Delivered food and drink from restaurants and food outlets to people  3. Provided courier services (e.g. package and postal deliveries, messenger services, etc.)  4. Performed manual tasks (e.g. cleaning, decorating, building, home fixtures and repairs, pet-sitting, etc.)  5. Performed non-manual tasks (e.g. web and software development, writing and translation, accounting, legal and admin services, marketing and media, audio and visual services, etc.)  96. None of these | Respondents selecting any of options 1–5 are classified as engaging in gig work. Respondents selecting “None of these” (96) are classified as not engaging in gig work. | Our operationalization is consistent with prior literature in defining gig work as participation in platform-mediated, income-generating activities and in distinguishing between primary dependence on gig work and supplementary engagement alongside other employment.  Guo et al. (2025) measure gig work using UKHLS questions asking whether respondents “have done any of the following jobs using a website, platform, or app in order to make money during the last month,” and distinguish between “high-dependence gig workers” and “low-dependence gig workers” based on whether individuals rely solely on gig work. Similarly, Glavin and Schieman (2022) define platform work engagement based on whether respondents “used an app-based or web-based service to earn income,” and differentiate between those who consider platform work their “main job” and those who engage in it as a secondary activity. Consistent with this approach, Lu et al. (2023) use UKHLS data classify gig work based on participation in platform-mediated activities and define gig workers as those relying solely on such work when comparing them with formal employment. |
| Waves 13–14 | What type(s) of work do you get using these websites or digital platforms? (CODE ALL THAT APPLY) | 1. Driving/taxi services  2. Delivery/courier services  3. Professional work (e.g., consultancy, legal, accounting)  4. Creative or IT work  5. Administrative work  6. Skilled manual work  7. Personal services  8. Selling own goods  9. Reselling goods  10. Renting property  97. Other  96. None of these | Respondents selecting any category except “None of these” (96) are classified as engaging in gig work. |  |
| Employment status (all waves) | Are you an employee or self-employed? | 1. Employee  2. Self-employed | Combined with gig work:  • High-dependence gig work: gig work + no other employment  • Low-dependence gig work: gig work + employee/self-employed |  |

| **Table A6**  **Random effect regression on chronic illness and psychological distress (Gig works in employment arrangements are categorized by proportion of gig work hours)** | | | | |
| --- | --- | --- | --- | --- |
|  | Model 1 | Model 2 | Model 3 | Model 4 |
|  | Total | Total | Men | Women |
| Chronic illness (ref.=Without chronic illness) |  |  |  |  |
| With chronic illness | 1.439^***^ | 1.511^***^ | 1.349^***^ | 1.508^***^ |
|  | (0.039) | (0.051) | (0.053) | (0.056) |
| Gender (ref.=Female) |  |  |  |  |
| Male | -0.580^***^ | -0.539^***^ |  |  |
|  | (0.033) | (0.038) |  |  |
| Chronic illness * Gender (ref.=Female) |  | -0.166^*^ |  |  |
|  |  | (0.076) |  |  |
| Partnership status (ref.=No) |  |  |  |  |
| Yes | -0.380^***^ | -0.378^***^ | -0.073 | -0.535^***^ |
|  | (0.041) | (0.041) | (0.066) | (0.054) |
| Race (ref.=Non-white) |  |  |  |  |
| White | 0.160^***^ | 0.160^***^ | 0.087 | 0.218^***^ |
|  | (0.042) | (0.042) | (0.058) | (0.060) |
| Age | -0.030^***^ | -0.030^***^ | -0.030^***^ | -0.031^***^ |
|  | (0.001) | (0.001) | (0.002) | (0.002) |
| Presence of dependent children (ref.=No) |  |  |  |  |
| Yes | 0.258^***^ | 0.258^***^ | 0.291^***^ | 0.195^***^ |
|  | (0.035) | (0.035) | (0.049) | (0.049) |
| Educational attainment (ref.=Below tertiary) |  |  |  |  |
| Tertiary | 0.188^***^ | 0.188^***^ | 0.271^***^ | 0.110^*^ |
|  | (0.033) | (0.033) | (0.044) | (0.048) |
| Employment arrangement (ref.=Unemployment) |  |  |  |  |
| Regular employment | -0.634^***^ | -0.635^***^ | -0.703^***^ | -0.607^***^ |
|  | (0.033) | (0.033) | (0.047) | (0.046) |
| High-dependence gig work | 0.170 | 0.170 | 0.159 | 0.177 |
|  | (0.179) | (0.179) | (0.232) | (0.267) |
| Low-dependence gig work | -0.560^***^ | -0.561^***^ | -0.678^***^ | -0.485^***^ |
|  | (0.055) | (0.055) | (0.073) | (0.081) |
| Within R-squared | 0.005 | 0.005 | 0.005 | 0.006 |
| Observations | 75428 | 75428 | 33954 | 41474 |
| Number of respondents | 22712 | 22712 | 10121 | 12591 |
| Note: Standard errors are in parentheses. ***p < 0.001, **p < 0.01, *p < 0.05; ref. = reference category. | | | | |

| **Table A7**  **Moderating effect of employment arrangement (Gig works in employment arrangements are categorized by proportion of gig work hours)** | | | |
| --- | --- | --- | --- |
|  | Model 1 | Model 2 | Model 3 |
| Chronic illness * Employment arrangement (ref.= Regular employment) | Total | Men | Women |
| With chronic illness * Unemployment | 0.397^***^ | 0.308^***^ | 0.458^***^ |
|  | (0.068) | (0.093) | (0.097) |
| With chronic illness * High-dependent gig work | 0.759 | 1.523^**^ | 0.019 |
|  | (0.421) | (0.536) | (0.643) |
| With chronic illness * Low-dependent gig work | -0.257^*^ | -0.156 | -0.345 |
|  | (0.125) | (0.175) | (0.177) |
| Within R-squared | 0.005 | 0.005 | 0.006 |
| Observations | 75428 | 33954 | 41474 |
| Number of respondents | 22712 | 10121 | 12591 |
| Note: Standard errors are in parentheses. ***p < 0.001, **p < 0.01, *p < 0.05; ref. = reference category. Controlled for all covariates mentioned in Table 2. | | | |

| **Table A8**  **Random effect regression on health problems lasting at least 12 months and psychological distress** | | | | |
| --- | --- | --- | --- | --- |
|  | Model 1 | Model 2 | Model 3 | Model 4 |
|  | Total | Total | Men | Women |
| Health problems (ref.=Without health problems) |  |  |  |  |
| With health problems | 1.043^***^ | 1.174^***^ | 0.880^***^ | 1.177^***^ |
|  | (0.025) | (0.033) | (0.034) | (0.036) |
| Gender (ref.=Female) |  |  |  |  |
| Male | -0.605^***^ | -0.512^***^ |  |  |
|  | (0.031) | (0.034) |  |  |
| Health problems * Gender (ref.=Female) |  | -0.290^***^ |  |  |
|  |  | (0.049) |  |  |
| Partnership status (ref.=No) |  |  |  |  |
| Yes | -0.423^***^ | -0.420^***^ | -0.148^*^ | -0.570^***^ |
|  | (0.039) | (0.039) | (0.061) | (0.051) |
| Race (ref.=Non-white) |  |  |  |  |
| White | 0.105^**^ | 0.104^**^ | 0.011 | 0.176^**^ |
|  | (0.039) | (0.039) | (0.053) | (0.055) |
| Age | -0.027^***^ | -0.027^***^ | -0.027^***^ | -0.029^***^ |
|  | (0.001) | (0.001) | (0.002) | (0.002) |
| Presence of dependent children (ref.=No) |  |  |  |  |
| Yes | 0.275^***^ | 0.274^***^ | 0.302^***^ | 0.215^***^ |
|  | (0.032) | (0.032) | (0.046) | (0.046) |
| Educational attainment (ref.=Below tertiary) |  |  |  |  |
| Tertiary | 0.147^***^ | 0.148^***^ | 0.240^***^ | 0.064 |
|  | (0.031) | (0.031) | (0.041) | (0.045) |
| Employment arrangement (ref.=Unemployment) |  |  |  |  |
| Regular employment | -0.674^***^ | -0.674^***^ | -0.722^***^ | -0.661^***^ |
|  | (0.031) | (0.031) | (0.044) | (0.043) |
| High-dependence gig work | 0.087 | 0.084 | 0.272 | -0.110 |
|  | (0.172) | (0.172) | (0.218) | (0.263) |
| Low-dependence gig work | -0.590^***^ | -0.592^***^ | -0.701^***^ | -0.519^***^ |
|  | (0.052) | (0.052) | (0.069) | (0.076) |
| Within R-squared | 0.006 | 0.006 | 0.005 | 0.008 |
| Observations | 83159 | 83159 | 37787 | 45372 |
| Number of respondents | 25879 | 25879 | 11664 | 14215 |
| Note: Standard errors are in parentheses. ***p < 0.001, **p < 0.01, *p < 0.05; ref. = reference category. | | | | |

| **Table A9**  **Moderating effect of employment arrangement (Independent variable = Health problems lasting at least 12 months)** | | | |
| --- | --- | --- | --- |
|  | Model 1 | Model 2 | Model 3 |
| Health problems * Employment arrangement (ref.= Regular employment) | Total | Men | Women |
| With health problems * Unemployment | 0.338^***^ | 0.335^***^ | 0.330^***^ |
|  | (0.048) | (0.066) | (0.069) |
| With health problems * High-dependent gig work | 0.645 | 1.685^***^ | -0.251 |
|  | (0.352) | (0.458) | (0.530) |
| With health problems * Low-dependent gig work | 0.007 | -0.062 | 0.058 |
|  | (0.101) | (0.136) | (0.147) |
| Within R-squared | 0.006 | 0.006 | 0.008 |
| Observations | 83159 | 37787 | 45372 |
| Number of respondents | 25879 | 11664 | 14215 |
| Note: Standard errors are in parentheses. ***p < 0.001, **p < 0.01, *p < 0.05; ref. = reference category. Controlled for all covariates mentioned in Table 2. | | | |
